# Variability in Cancer Risk and Outcomes Within US Latinos by National Origin and Genetic Ancestry

**DOI:** 10.1007/s40471-016-0083-7

**Published:** 2016-06-30

**Authors:** Mariana C. Stern, Laura Fejerman, Rina Das, V. Wendy Setiawan, Marcia R. Cruz-Correa, Eliseo J. Perez-Stable, Jane C. Figueiredo

**Affiliations:** 1 Department of Preventive Medicine, Keck School of Medicine, University of Southern California, Los Angeles, CA USA; 2 Department of Urology, Keck School of Medicine, University of Southern California, Los Angeles, CA USA; 3 Norris Comprehensive Cancer Center, Keck School of Medicine, University of Southern California, Los Angeles, CA USA; 4 Department of Medicine, Division of General Internal Medicine, University of California San Francisco, San Francisco, CA USA; 5 National Institute on Minority Health and Health Disparities, National Institutes of Health, Bethesda, MD USA; 6 Department of Medicine and Biochemistry, University of Puerto Rico Medical Sciences Campus and University of Puerto Rico Comprehensive Cancer Center, San Juan, Puerto Rico

**Keywords:** Cancer, Hispanic, Latino

## Abstract

Latinos have lower rates for most common cancer sites and higher rates of some less common cancers (gallbladder, liver, gastric, and cervical) than other ethnic/racial groups. Latinos are a highly heterogeneous population with diverse national origins, unique genetic admixture patterns, and wide spectrum of socio-demographic characteristics. Across the major cancers (breast, colorectal, prostate, lung, and liver) US-born Latinos have higher incidence and worse survival than foreign-born, and those with low-socioeconomic status have the lowest incidence. Puerto Rican and Cuban Latinos have higher incidence rates than Mexican Latinos. We have identified the following themes as understudied and critical to reduce the cancer burden among US Latinos: (1) etiological studies considering key sources of heterogeneity, (2) culturally sensitive cancer prevention strategies, (3) description of the molecular tumor landscape to guide treatments and improve outcomes, and (4) development of prediction models of disease risk and outcomes accounting for heterogeneity of Latinos.

## Introduction

Hispanics/Latinos (referred henceforth as Latinos) are the largest and fastest growing minority group in the USA, and with a population of over 55 million, they account for 17 % of the US population in 2014 [1], which is predicted to increase to 35 % by the year 2050. Despite having higher poverty rates, lower education, and less access to health care than non-Hispanic Whites (NHW), Latinos tend to have overall better health indicators than those of other racial/ethnic groups with whom they share socio-economic (SES) characteristics. This epidemiologic phenomenon coined “the Hispanic paradox” [2–5] might be explained by cultural and lifestyle practices brought by Latino immigrants from their countries of origin, reproductive behavior, extended family support, and a distinct genetic heritage [3]. It has been proposed that these putative health-related advantages in Latino immigrants may erode over successive generations in the USA with increased assimilation [4]. Overall, cancer incidence rates (breast, colorectal, lung, and prostate) in US Latinos tend to be higher than those reported for most Latin American countries [6•], although differences in completeness of cancer registries between the USA and Latin America cannot be discarded as a possible explanation. Mortality rates also tend to be lower for Latinos compared to NHW; however, there are substantial disparities in cancer prevalence, care, and outcomes that are still not understood.

Latinos are the result of more than 500 years of admixture of European, Indigenous American (IA), and African individuals, with varying degrees across Latin America [7]. In the USA, the majority of Latinos are of Mexican national origin (64.3 %), followed by Puerto Rican (9.5 %), Salvadorean (3.7 %), Cuban (3.7 %), and Dominican (3.1 %), with the rest coming from other Central and South American countries [8]. Overall, about 60 % of these individuals are US-born, with the remaining 40 % born in Mexico (64 %), Puerto Rico (9 %), and other Latin American countries. US Latinos include recent immigrants who make similar lifestyle and dietary choices as those in their countries of origin, as well as second or higher generation immigrants born in the USA who are partially or fully assimilated to the US lifestyle. Both genetic ancestry and nativity correlate with known cancer determinants. For example, there are differences in trends for body mass index (BMI) by nativity among US Latinos, with US-born Mexican and Puerto Ricans having greater annual increases in BMI than US-born Cubans and foreign-borns [9]. Also, Latinos of higher IA or African ancestry are more likely to have a lower SES than those with higher European ancestry [10–12].

This heterogeneity presents a unique opportunity to disentangle the complex role of SES, culture, lifestyle, and genetics, as potential determinants of cancer risk in Latinos and other populations. In spite of this rich diversity, epidemiological studies have largely ignored it and have grouped Latinos as a single ethnic group. A better understanding of the heterogeneity that exists within Latinos may give important clues regarding the key cancer determinants and cancer characteristics in this population and help achieve the goal of personalized medicine in this fast growing minority group.

## Overall Cancer Risk Patterns Among Latinos

Overall, Latinos in the USA have lower incidence rates for the most common cancer sites, namely breast, lung, colorectal, and prostate cancer compared to NHW and African Americans (AA). However, they have higher incidence rates for cervical, penile, and some gastrointestinal cancers (gallbladder, gastric, and liver), which are typically associated with low SES, obesity, and diabetes and many of them also with infections (e.g., Human Papillomavirus, hepatitis B and C viruses, and *Helicobacter pylori*). Additionally, compared to NHW, and similarly to AA [6•], Latinos are more likely to be diagnosed at an advanced stage of disease for most common cancers and have higher mortality rates for select cancers such as breast, gastrointestinal, uterine, and cervical, potentially due to lower rates of access to care, limited proper screening, lack of early detection, and/or biologic factors. An increasing trend of early-onset disease (<50 years) among Latinos in the last few decades has been reported [13–15, 16•, 17]. Importantly, these trends in incidence and mortality differ widely among Latino subpopulations.

## Variability in Cancer Determinants Among Latino Subpopulations

As with other racial/ethnic populations in the USA, differences in cancer determinants across US Latinos are in part due to substantial variation within this population in the prevalence of well-established cancer risk factors such as smoking, low quality diet, and physical inactivity. In addition, limited access to health care and financial constraints among US Latinos has been associated with lower cancer screening rates [18, 19]. Even though Latinos share a common language and history, similarly to other migrant populations in the USA, such as Asians, there is significant variability in cultural factors, which vary by countries of origin, nativity, and level of assimilation to the USA lifestyle. For foreign-born Latinos, there is also further variability in exposures acquired in their countries of origin and the social class of origin. The degree to which individuals have assimilated to the USA and their SES strongly influence behavioral patterns related to cancer prevention, diagnosis, and treatment [20, 21].

Unlike other populations, among Latinos, there is the added variability introduced by their admixed genetic background. The proportion of any one of the three main ancestral populations could affect the presence of cancer susceptibility alleles and/or could be a proxy for other factors that might be tightly correlated with ancestry, such as SES, culture (e.g., adherence to traditional values), and lifestyle. These factors could jointly or independently determine cancer risk and survival. We summarize below the existing knowledge on cancer trends, risk factors, clinical characteristics, and survival patterns among Latinos for selected cancers (breast, colorectal, prostate, lung, and liver) for which there is available evidence of disparities across subpopulations defined by nativity, genetic ancestry, and/or other sources of diversity within Latinos.

## Breast Cancer

### Incidence

Breast cancer (BC) is the most common cancer in US Latinas, with 19,800 women expected to be diagnosed in 2015 [1]. However, within Latinas, incidence varies substantially by national origin [6•], place of birth (US-born vs. foreign-born) and neighborhood context [22, 23]. In Florida, Puerto Rican and Cuban Latinas have higher rates of BC than those from Mexico and other Latin American countries combined, comparable to those in AA [6•]. Puerto Rican women living on the island have been reported to have a significantly lower incidence rate than those living in mainland USA [24], and foreign-born Latinas in California have lower risk of developing BC than US-born [22], with increasing risk with longer US residency [23]. The 2000 and 2005 National Health Interview Survey (NHIS) Cancer Control Modules observed a higher 5-year absolute risk in Cubans/Cuban Americans compared to Mexican/Mexican Americans and a higher lifetime risk in Dominicans compared to Mexican/Mexican Americans [25].

Studies focused on genetic ancestry reported that Latinas with high IA ancestry have lower risk of developing BC than those with high European ancestry, which was still observed after controlling for most established risk factors that are known to differ between Latina and NHW women [10, 26]. Moreover, Latina women who live within neighborhoods with mostly Latinos and with high use of Spanish language (“Hispanic enclaves”) and with low SES have lower risk than their counterparts [22]. The role of African ancestry in defining BC risk by Latina national origin has not been examined.

### Risk Factors

Exposure to anthropometric, reproductive, behavioral, and genetic factors differs between Latino subpopulations, which might contribute to observed differences in incidence. For example, Latinas with higher IA ancestry tend to have more children than those with higher European ancestry [27]. BMI is associated with genetic ancestry among Latinas, although the direction of association is inconsistent between studies from different US regions [27, 28], suggesting an additional source of heterogeneity in subpopulation differences in BC-related exposures. However, consideration of these factors still shows an independent effect of IA ancestry on BC risk [10, 26], suggesting there are additional lifestyle or behavioral factors that correlate with IA ancestry yet to be discovered, and/or genetic risk factors.

Multiple candidate gene studies have reported heterogeneity in associations by IA ancestry [29–32]. A GWAS study of BC in Latinas found a protective variant near the *ESR1* gene that is only observed in women that have IA ancestry. The frequency of the protective variant in women of different national origins correlates with the known average proportion of IA ancestry for the region: It has a frequency of 5 % in Puerto Ricans, 10 % in Colombians, 14 % in Mexicans, and 23 % in Peruvians [33•]. This study also confirmed the association of previously reported variants, with 84 % of the polymorphisms having odds ratios that were directionally concordant with those reported in European and/or Asian studies [33•].

### Clinical Characteristics and Survival

In California, Latinas living in low SES and high “Hispanic enclave” neighborhoods were ∼24 % more likely to be diagnosed with more advanced stage than their counterparts [34]. Being foreign-born was an independent predictor of higher stage at diagnosis; however, despite this, foreign-born Latinas had marginally better survival than US-born Latinas, independently of SES and neighborhood enclave, stage, and treatment [34]. When considering genetic ancestry, California Latinas with more than 50 % IA ancestry have approximately twice the mortality hazard of women with 50 % or less [35], and Caribbean women had a higher mortality hazard than women from Central America, after controlling for genetic ancestry, education, SES, tumor characteristics, and treatment information [35]. Genetic ancestry was not found to be associated with tumor characteristics (i.e., estrogen and progesterone receptor status, stage and grade at diagnosis, and tumor size) or type of treatment received in the first fourth months following diagnosis [35].

## Colorectal Cancer

### Incidence

Colorectal cancer (CRC) is the second most common cancer in both Latino men and women, with an estimated total of 11,700 new cases and 3800 deaths in 2015 [1]. CRC incidence and death rates among Latinos are approximately 10 to 20 % lower than those among NHWs. However, incidence rates for CRC are higher among higher SES Latinos (68.1 per 100,000 for men and 43.4 per 100,000 for women) than among lower SES Latinos (41.5 per 100,000 and 28.7 per 100,000 for women) [36]. This pattern is the opposite of what is observed for NHW and AA. These data suggests that as Latinos acquire higher SES and assimilate to the US lifestyle, they may acquire additional risk factors that increase their CRC risk.

CRC incidence rates also vary substantially across Latinos by national origin. In Florida, CRC incidence rates among Cuban and Puerto Rican men were reported to be similar to those of NHW and twice those of Mexican men [6•]. Moreover, Mexican, Cuban, and Puerto Rican Latinos living in Florida had higher incidence rates than those living in their countries of origin or Puerto Rico [6•]. A separate study also reported that Puerto Ricans living in the island were found to have lower incidence rates than those living in various regions of mainland USA [24]. In California, Cuban Latinos had higher incidence than Mexican Latinos [37•]. In this same study, proportional incidence ratios considering SES and nativity showed that Cuban Latinos (both men and women) and Puerto Rican Latinas had more cases than expected, whereas Mexican Latinos had fewer cases than expected, confirming possible differences by national origin [37•].

Notably, incidence rates are increasing among adults aged younger than 50 years [14, 38]. In Puerto Rico, close to 10 % of all CRC cases are younger than 50 and two thirds of cases present at advanced stage. In response to this, the Puerto Rico Department of Health issued guidelines in 2015 to begin screening at age 40, and a national program using fecal immunological testing was established.

### Risk Factors

High intake of red meat and processed meats, lack of dietary fiber, alcohol intake, smoking, lack of physical activity, diabetes, family history of CRC, and obesity are known CRC risk factors. In the Multiethnic Cohort Study (MEC), Latinos reported a higher prevalence of obesity and diabetes, and lower prevalence of family history of CRC, colorectal polyps, and smoking, and higher consumption of dietary fiber, calcium, and folate than NHW [39]. Heritability estimates for CRC are approximately 12–35 %; however, highly penetrant, inherited mutations account for less than 5 % of these cancers. GWAS of CRC conducted mostly among NHW and Asians have identified 58 susceptibility alleles across 37 regions [40]. A study among Colombians reported a positive association between African ancestry and CRC risk [41]. In addition, the only GWAS study in US-Latinos in California reported that only a third of the known GWAS-identified risk loci in NHW were nominally statistically significant among Latinos; importantly, secondary susceptibility variants at 3q26.2 and 11q12.2 were identified in this population [42].

### Clinical Characteristics and Survival

Latinos tend to be diagnosed at a younger age and with higher stage, and Mexican Latinos in California were reported to have the greatest proportion compared to other Latino subgroups [43]. Moreover, Mexican Latinos have higher proportion of rectal cancer cases compared to other Latinos and NHW [43]. Recent studies have suggested a lower incidence of tumors with high microsatellite instability (MSI-High) and hereditary CRC syndromes in Latinos as compared to NHW [13, 44, 45], which is associated with better survival [46]. There is paucity of data on other tumor markers in Latinos, as well as knowledge of genetic variants that can be used for prognosis, all of which are critical for treatment decisions.

Latinos have improved survival rates compared to other ethnic/racial groups. However, US Latinos living in neighborhoods with more than 60 % Latinos had a 14 % greater chance of being diagnosed with late versus early stage CRC than Latinos living in neighborhoods with less than 20 % Latinos [47]. Furthermore, Latinos that live in low SES neighborhoods had lower survival than those living in high SES neighborhoods [48]. This association was more pronounced among US-born Latinos, who were found to have lower survival than foreign-born Latinos [48]. A “healthy immigrant” effect has been proposed as an explanation, but it does not seem to explain fully the observed disparities.

## Prostate Cancer

### Incidence

Prostate cancer (PC) incidence among Latino men is lower than among NHW or AA men; however, PC is the number one cancer that affects Latino men, with 13,000 men diagnosed in 2015 [1]. Mexican Latinos have been reported to have a lower incidence than NHW, Cubans and Puerto Rican Latinos [6•, 49, 50]. In contrast, Cuban and Puerto Rican Latinos have comparable or slightly higher rates than NHW [6•, 49, 50]. A study in Florida also reported that Mexican, Puerto Rican, and Cuban US Latinos had higher incidence rates than those reported in Mexico, Cuba, and Puerto Rico [6•]. Moreover, Puerto Ricans living in the island have lower cancer incidence rates than those living in various regions of the mainland US [24] or US Latinos [51].

Latinos living in the highest SES category were reported to have an incidence rate that was 78 % higher than those living in the lowest SES category [36]. Whereas similar disparities have been observed for NHW, AA, and Asians, those observed for Latinos were considerably greater. PSA testing is lower among Latinos compared to other racial groups [52], and more commonly used among higher SES men. However, differences in PSA use are unlikely to explain fully the observed disparity by SES as this disparity is also observed when restricting cases to aggressive cancer, which are less likely to be detected by PSA screening [49].

### Risk Factors

There are few established risk factors for prostate cancer other than age, family history of PC and race/ethnicity, and even fewer uniquely identified for Latinos. Several common genetic variants have been identified through GWAS studies that can increase the risk of PC among NHW and AA men; many of these have been reported to be associated with risk among Latinos [53]. A study among Mexican Latinos in Texas identified associations between exposure to agrichemicals, being US-born, lack of physical activity and PC family history and higher risk of PC [54]. This finding is supported by the observation that California farm workers, which are predominantly Mexican migrant workers, showed disproportionately more PC cases than Californian NHW [55]. Diets high in red meat have been proposed to increase PC risk, and an association between red meat cooked at high temperatures and risk was reported among Latinos in a multi-ethnic study [56]. Interestingly, Latinos showed higher prevalence of intake of red meat cooked using high temperature methods, and whereas no associations were found for specific meat mutagens and NHW and AA men, significant positive associations were reported for Latinos [56].

### Clinical Characteristics and Survival

Unlike NHW and AA men, Latino men in Texas with higher degree of SES deprivation have reduced mortality compared to men with low deprivation [57]. Moreover, in California, foreign-born Latino men were reported to be more likely to be diagnosed with advanced disease than US-born men; however, those who resided in high Hispanic enclave neighborhoods, in spite of the overall lower SES, had better survival than US-born Latinos living in comparable neighborhoods [58]. In contrast with these findings, mortality rates were reported to be higher among Puerto Rican men living in the island compared to mainland US Latinos [51].

## Lung Cancer

### Incidence

Lung cancer (LC) is the third-most commonly diagnosed cancer in Latino men and the fifth most common in Latinas. Incidence rates (43.4 and 26 per 100,000 for men and women, respectively) are lower than in NHW and AA, with 9600 Latinos expected to be diagnosed in 2015 [59•]. However, differences in incidence have been reported by nativity. Incidence rates are reported to be lowest among Mexican Latinos, followed by Puerto Ricans and Cubans [50]. Moreover, Puerto Ricans living on the island have lower incidence rates than Puerto Ricans living in Florida [6•, 24]. Similar to other cancers, and in complete contrast with NHW, AA, and Asians, incidence rates for Latinos in California were highest among high SES and lowest among low SES [36].

### Risk Factors

The observed heterogeneity in LC rates within the Latino population may reflect differences in smoking patterns. The overall percentage of smokers among Latino men and women is lower than for NHW or AA [60, 61], with rates being higher among US-born Latinos than among foreign-born, especially among women. Cubans and Puerto Ricans are more likely to smoke (18 %) than Mexicans (13 %), Central Americans, or Dominicans [1, 60, 62, 63]. In addition, Latino smokers of all national origins tend to smoke less intensively than NHW with about 50 % smoking 5 or fewer cigarettes per day or reporting non-daily smoking [64]. Interestingly, given similar smoking intensity, Latinos in the MEC Study were shown to have 30 to 70 % lower relative risk of developing LC than the AA reference group until smoking intensity reached 30 cigarettes per day [65]. Non-smoking Latinos have low levels of second-hand smoke exposure overall, but Latinos tend to have higher exposure at their worksite [66]. A study in New Mexico reported that Latinos were more likely than NHW to have a composite methylation index in sputum, reported to associate with LC risk, and a more rapid increase in risk was observed as a function of cigarette smoke [67•]. However, among Latinos, higher IA ancestry was associated with lower risk of detecting this methylation index [67•].

### Clinical Characteristics and Survival

Among Latinos, LC is the leading cause of cancer death in men (17 %) and the second leading cause of cancer death in women (13 %) [59•]; however, the mortality rates are 50 % those of NHW [1]. Among women, Cubans (13.4 %) and Puerto Ricans (13.3 %) have a higher percentage of death due to LC compared to other Latinas (11.9 %) [68]. Among men, the proportion of cancer deaths were higher for Cuban men (30.1 %) followed by Mexican (23.2 %) and Puerto Rican (23.7 %) men [68]. Also, Latino men have a 5-year survival rate of 15 %, which is lower than the 18 % reported for NHW; whereas among women rates are comparable (23 vs. 24 %, NHW and Latinas, respectively). However, Latinos, both US- and foreign-born, are reported to have improved overall survival compared to NHW [69]. This overall survival advantage of Latino patients with non-small cell lung cancer (NSCLC) might be because of higher prevalence of histologic subtypes (e.g., bronchioloalveolar carcinoma) of NSCLC associated with better prognosis [69]. Despite the fact that Latinos are more likely than NHW to be diagnosed at a distant stage of disease (58 vs. 54 %) [1], with higher pathologic stage, they are less likely to receive curative treatment [70].

The molecular status of several tumor (“driver”) mutations is often assessed in order to select the most appropriate treatment for LC, and successful therapies have been developed to target *EGFR* mutations or EML4-ALK fusions [71]. The prevalence and characteristics of these driver mutations differ among racial and ethnic groups [72]. Specifically, whereas *KRAS* mutations are predominant in NHW, among Latinos *EGFR* mutations are more frequent [71, 73, 74]. Variability in *EGFR* and *KRAS* mutation frequency in NSCLC has been reported for Latinos from various countries in Latin America, with the highest frequency observed in Peru, followed by Costa Rica, Mexico, Panama, Colombia, and Argentina [72, 73]. Exposure to wood smoke and *Mycobacterium tuberculosis* infection have been found to associate with EGFR mutations and proposed as potential causal factors [72].

## Liver Cancer

### Incidence

From 2008 to 2012, rates of liver cancer were 19.3 per 100,000 in men and 7.2 per 100,000 in women; these rates are higher than NHW and AA, but lower than Asians and IA [59•]. While the rates of most cancers in Latino populations are declining, the rate of hepatocellular carcinoma (HCC), the most common type of liver cancer, is increasing [75]. In 1999–2001, the liver cancer mortality rates among US-born Latino men were more than twice as high than those for foreign-born Latino men in California and 65 % higher in Texas [76]. In California, between 1988 and 2004, US-born Latino men in California had 86 % higher incidence rates than foreign-born Latino men [77]. In a recent study in the MEC, the HCC incidence rate (per 100,000) was almost twice as high in US-born as in foreign-born Latino men (44.7 vs. 23.1), but comparable and much lower in women (14.5 vs. 13.4) [78]. Two other studies showed higher incidence rates among Latino men in Florida compared to the incidence rates in their country of origin [6•] and higher rates in US mainland than island Puerto Rican men, but not in women [24]. Within the Latino subpopulation in Florida, Puerto Rican men had the highest incidence rate compared to the Mexicans and Cubans [6•]. In California, foreign-born Latinos had lower incidence rates than US-born Latinos, and those living in low SES and high enclave neighborhoods had higher incidence rates than their counterparts [77]. However, it was noted that nativity seemed to be a more important risk determinant than type of neighborhood.

### Risk Factors

Known risk factors for HCC include chronic hepatitis B (HBV) and hepatitis C (HCV) infection, aflatoxin exposure, excessive alcohol consumption, obesity, diabetes, cirrhosis of any etiology, and smoking. Coffee drinking has been associated with lower HCC incidence [79]. Among Latinos, studies have suggested that HCV and diabetes are the strongest risk factors for HCC [78]. It has been shown that eliminating diabetes could potentially reduce HCC incidence in all ethnic/racial groups, with the largest potential for reduction in Latinos [80]. Another important emerging risk factor that has been associated with HCC incidence is non-alcoholic fatty liver disease [81].

A greater prevalence of HCC risk factors among the more acculturated US-born Latinos is likely to explain the disparity in HCC incidence by nativity [76]. Acculturation has been associated with increased rates of smoking, alcohol drinking, and obesity in Latinos [82, 83]. In the MEC, US-born Latinos had higher rates of smoking, alcohol drinking, and obesity compared to foreign-born Latinos. The diverging nativity patterns by sex for HCC might be due to a stronger tendency of men to adopt unhealthy lifestyles (e.g., excessive alcohol drinking, weight gain, and illicit drug use) compared with women [6•]. In the MEC, the difference in the prevalence of HBV/HCV and diabetes between US-born and foreign-born Latinos is larger in men than in women [78]. Thus, the greater difference in prevalence of these factors between the US- and foreign-born Latino men than women could partially explain the observed HCC incidence difference in men, but not in women, between the two nativity groups. US-born Latinos in California were reported to have higher prevalence of binge drinking than foreign-born Latinos (California Health Interview Survey, CHIS). However, even after accounting for differences in known risk factors, the risk of HCC among US-born Latinos remained significantly higher compared to the risk among foreign-born Latinos [78], suggesting that future studies are warranted to identify additional risk factors that contribute to the elevated HCC incidence in US-born Latinos.

HCV is more common among Puerto Ricans than other Latino groups [84], whereas heavy alcohol drinking is more common in Mexican and Puerto Rican men [85], which could contribute to the observed differences in HCC incidence rates across subgroups. Finally, exposure to aflatoxins, mycotoxins and known liver carcinogens present in moldy foodstuffs, might play a role in Mexican and Central American Latinos [86].

### Clinical Characteristics and Survival

At the time of diagnosis, Latinos are more likely to be diagnosed at a localized stage compared with other ethnic/racial groups [87]. Despite the low disease stage, Latinos were 25 % less likely to receive local ablation or surgical resection compared to NHW; less access to care, lower SES, as well as language/cultural barriers were thought to contribute to this difference in treatment [88]. HCC is one of the most fatal cancers; the 5-year survival rate among Latinos is 19 % for both men and women [89]. Between 1987 and 2001, Latinos were shown to have lower survival relative to NHW [87]. Receipt of therapy, stage of HCC, year of diagnosis, and other demographics, however, did not explain the increased mortality risk among Latinos. Further research is needed to understand if tumor characteristics and prognosis differs by ancestry, birth location and socioeconomic factors.

## Cancer Research in Latinos: Public Health and Clinical and Translational Impact

Despite immense diversity within the US Latino community in terms of nativity, genetic ancestry, and degree of acculturation, epidemiological studies tend to consider US Latinos as one single group. The emerging evidence for some of the major cancer types (breast, colorectal, prostate, lung, and liver) highlights patterns observed across Latino subpopulations defined by various indicators of heterogeneity. US-born Latinos have higher cancer incidence than foreign-born Latinos. Puerto Rican and Cuban Latinos have higher incidence rates than Mexican Latinos. Opposite to other racial groups, Latinos living in low SES neighborhoods tend to have lower incidence rates than those living in higher SES, and for some cancers (breast), lower incidence rates are also seen in high Latino enclave neighborhoods. Foreign-born Latinos tend to have better survival compared to US-born Latinos. Overall, the emerging picture is that lifestyle and behavioral factors preserved or fostered among Latino immigrants can lower cancer risk. One study investigating the role of genetic ancestry in breast cancer suggested that genetic variants of IA origin might lower cancer risk, highlighting the possibility that this could be true for other cancers. We summarize below the understudied themes that emerge as more pressing to advance our knowledge of cancer in Latinos and reduce their cancer burden.

### Understanding Risk Profiles and Developing Prediction Models

There is a dearth of knowledge about risk factors specific for Latinos and their variability across Latino subpopulations. Given that by the year 2050, close to 1 in 4 people in the USA will be Latino, it is critical that we identify the main cancer determinants in this population. The observed disparities across subpopulations, and the fact that Latinos are a population in transition, offer a tremendous opportunity to identify risk factors and reduce future disease rates. For prevention strategies to be successful, they will need to be culturally appropriate, as determinants of compliance with preventive strategies might differ from those established for other US racial/ethnic groups.

There is emerging interest on the potential value of genetic risk profiling for screening for selected cancer sites, namely breast and colorectal. Many of the GWAS loci identified in NHW and Asians appear to generalize to Latinos, although some studies indicate that there may be “better” markers for those genetic regions or other SNPs that may prove more informative. Existing risk predictive models, such as the widely used Gail model for breast cancer risk, have been systematically used in Latinas. However, it is likely that prediction would improve if models were developed specifically in Latina populations and not just generalized from those developed in NHW populations. Understanding these potential ethnic/racial differences is pressing.

### Tailored Treatment, Reducing Disparities and Improving Prognosis

Albeit scarce, data is emerging that Latinos may harbor tumor somatic mutations with different prevalence than other racial groups. Given that cancer treatments are moving towards the goal of personalized medicine, understanding the tumor landscape of Latinos will be essential to identify the most adequate therapies. In these studies, it will be imperative to account for genetic ancestry, and scientific successes in doing so are already apparent. For example, higher IA ancestry is associated with Acute Lymphoblastic Leukemia (ALL) relapse among Latino children, even after adjustment for prognostic factors [90•]. ALL is the most common type of cancer in children and disproportionally affects Latinos [91, 92], who have worse treatment outcome than NHW [92–94]. Higher prevalence of germline variants in the *GATA3* gene are linked to higher risk of relapse among Latino children [95]. Incorporation of an additional phase of chemotherapy among patients with high IA ancestry drastically reduced the disparity in prognosis [90•]. Given that IA or African ancestry in Latinos also correlates with SES and sociocultural factors, which can also affect prognosis (e.g., patterns and quality of care) [92], both genetic and non-genetic heterogeneity are likely to contribute to ALL outcomes in Latinos and should be considered jointly. A similar scenario may apply to other cancers and deserves further investigation.

## Conclusions

Variability in genetic ancestry, exposures, cultural values brought or inherited from the countries of origin, and the degree of assimilation to US lifestyle are major contributors of heterogeneity among Latinos. This heterogeneity has an impact on cancer risk, tumor characteristics, and outcomes. As the focus on Latino cancer research increases, elucidating the complex interactions between the different sources of heterogeneity in this understudied population will be critical in order to guide primary prevention, diagnosis, and treatment strategies tailored for Latinos. To do so, improving the completeness of country of origin data in cancer registries will be essential. For this to happen, it will be important to collect more detailed information on Latino subpopulations on hospital admission records at the time of diagnosis. Moreover, it would help if funding agencies also requested more detailed classification of Latinos than simply obtaining a single ethnic category. This will help emphasize to the public health and research community the need to address heterogeneity within Latino subpopulations with respect to disease risk and outcomes.

### Disclaimer

The findings and conclusions in this article are those of the authors and do not necessarily represent the views or the official position(s) of the National Institutes of Health or any of the sponsoring organizations and agencies of the US government.
